# How Motives Related to Benefits for Oneself and Others Would Affect COVID-19 Vaccination in a Hong Kong Chinese General Adult Population?

**DOI:** 10.3390/vaccines10111883

**Published:** 2022-11-08

**Authors:** Yanqiu Yu, Mason M. C. Lau, Joseph T. F. Lau

**Affiliations:** 1 Department of Preventive Medicine and Health Education, School of Public Health, Fudan University, Shanghai 200032, China; 2 Key Laboratory of Public Health Safety, Fudan University, Shanghai 200032, China; 3 Centre for Health Behaviours Research, Jockey Club School of Public Health and Primary Care, The Chinese University of Hong Kong, Hong Kong, China; 4 Zhejiang Provincial Clinical Research Center for Mental Disorders, The Affiliated Wenzhou Kangning Hospital, Wenzhou Medical University, Wenzhou 325000, China; 5 School of Mental Health, Wenzhou Medical University, Wenzhou 325000, China; 6 School of Public Health, Zhejiang University, Hangzhou 310058, China

**Keywords:** COVID-19 vaccination, health behaviour, general population, outcome expectancy, prosociality

## Abstract

Outcome expectancies involving self-directed and others-directed domains are potential determinants of completed or scheduled first-dose COVID-19 vaccination (CSFCV). This study investigated factors of CSFCV, including (a) self-directed motives [personal positive outcome expectancies (POE) and personal negative outcome expectancy (NOE)], and (b) others-directed motives (societal POE and the personality trait of prosociality). It also investigated the mediations of personal POE between societal POE and CSFCV, and moderations of prosociality between personal POE/personal NOE/societal POE and CSFCV. A cross-sectional population-based telephone survey interviewed 500 people aged 18–75 in Hong Kong in May 2021. The prevalence of CSFCV was 21.0%. Significant factors of CSFCV included personal POE (i.e., physical/practical/emotional/interpersonal benefit), personal NOE, and societal POE. The association between societal POE and CSFCV was fully mediated by the overall scale and some domains of personal POE. Furthermore, the association between physical benefit and CSFCV was stronger at lower levels of prosociality; prosociality showed a stronger effect on CSFCV at lower levels of physical benefit. The results suggest that self-directed motives might be more important than others-directed motives in affecting CSFCV. The findings require confirmations from longitudinal studies and cross-country comparisons.

## 1. Introduction

COVID-19 has been mentioned as one of the global challenges of the 21st century [1]. Vaccination is the most important means to control the COVID-19 pandemic. It contributes to the development of community immunity [2,3] and can reduce the number of hospitalizations and deaths effectively [4,5]. Globally, free vaccination programs have been rolled out. In July 2021, the prevalence of COVID-19 vaccination was quite low in many countries, and was moderately high in countries such as Israel (63.4%), Chile (69.6%), the U.K. (69.2%), China (51.3%), and the U.S. (56.0%) [6]. As of September 2022, COVID-19 vaccination rates have increased and exceeded 80% in some countries (e.g., China and Canada), but remained suboptimal in many places. For instance, only 67.0% of people in the U.S. and 52.5% in Russia have completed the primary vaccination series [6]. Given the high infectivity of COVID-19, very high COVID-19 vaccination rates are required to control the pandemic. A high vaccination coverage is also an important prerequisite for a country to relax COVID-19 control measures such as social distancing and travel restrictions [7]. Yet, vaccine hesitancy remained high throughout the entire rollout period [8,9]. Such hesitancy may retard motivation to take up booster doses. Health promotion is hence still warranted.

It is important to understand factors of COVID-19 vaccination in various phases of the rollout period so as to inform countries with low vaccination rates and in future pandemics. Reviews found that factors associated with COVID-19 vaccination included cognitive factors related to the pandemic and vaccination, emotional factors, trust towards the government, and interpersonal factors [8,10]. Among such factors, perceived benefits of COVID-19 vaccination is of key importance [11] as it provides motivation for vaccination [12].

This study investigated the associations between two types of motives (outcome expectations) related to perceived benefits and COVID-19 vaccination. Self-directed motives and others-directed motives refer to perceived outcomes of vaccination regarding oneself and other people, respectively. Literature shows that positive outcome expectancy (POE) and negative outcome expectancy (NOE) were associated with facemask wearing and handwashing [13], COVID-19 vaccination [14] and other types of vaccination (e.g., influenza vaccination) [15]. Outcome expectancy is a construct of both Social Cognitive Theory (SCT) [16] and Health Action Process Approach (HAPA) [17]; POE is similar to the construct of perceived benefit of the Health Belief Model (HBM) [12]. These theories commonly assume that people are rational when making health-related decisions, aiming at maximizing the benefits and minimizing the harms [16,17].

Self-directed motives include personal POE and NOE. Personal POE involves gains in the physical (e.g., protection), emotional (e.g., psychological relief), practical (e.g., convenience), and interpersonal (e.g., less interpersonal pressure under social norm) domains derived from COVID-19 vaccination. Personal NOE of COVID-19 vaccination mainly refers to the concerns about severe side effects (including death) due to vaccination, which were associated with vaccination hesitancy [18,19]. Others-directed motives is reflected by perceived societal POE (e.g., control of the pandemic, economic recovery, and removal of regional travel restrictions); such motives are important as COVID-19 hits countries’ economies badly and halts travelling. Although COVID-19 vaccination affects not only the vaccinated individuals but also society at large, most research considered only personal POE and NOE, but not societal POE of COVID-19 vaccination.

This study also tested whether the association between societal POE and COVID-19 vaccination would become non-significant (or remain significant but having a reduced magnitude) after adjusting for personal POE, i.e., it tested the hypothesis of full/partial mediation between societal POE and COVID-19 vaccination via personal POE. No previous research has looked at such relationships. The rationale of the mediation hypothesis is that a person may possess POE and NOE simultaneously, as societal benefits (e.g., control of the pandemic, recovery of the economy, and removal of travel restrictions) may result in personal benefits (physical, emotional, practical, and interpersonal gains). The reciprocal determinism construct of the SCT postulates that changes in the environment are associated with individual characteristics that may affect health behaviors [20]. As societal POE is related to changes in the social environment (e.g., relaxation of travel restriction policies), the SCT would imply a significant association between societal POE and personal POE, and supports the hypothesis that both societal POE and personal POE would be significantly associated with COVID-19 vaccination. Notably, the present study was unable and did not intend to test the SCT.

In addition, prosociality is related to ‘others-directed’ motives regarding COVID-19 vaccination. COVID-19 vaccination can be seen as a prosocial behavior [21,22]. Prosociality is defined as individuals’ enduring tendencies to enact behaviors such as sharing, helping, caring, and empathy [23]. Empirically, prosociality was positively associated with COVID-19 vaccination intention [24] and COVID-19 preventive behaviors (e.g., facemask wearing and social distancing) [25,26]. Experimental studies reported that increasing prosociality was able to enhance COVID-19 vaccination intention [27] and influenza vaccination [21,22]. Yet, no study has tested the association between prosociality and COVID-19 vaccination. Furthermore, prosociality may moderate the associations between POE/NOE and COVID-19 vaccination. These novel hypotheses regarding associations and moderations were tested in this study.

Contextually, all Hong Kong adult residents can make online appointments and choose to take up either free Pfizer-BioNtTech-Fosun or Sinovac Biotech vaccines at 29 conveniently located vaccination centers territory-wide. Incentives for COVID-19 vaccination include convenience to enter venues, relaxation in social distancing rules (e.g., size of table at restaurants), vaccination leaves, and ad hoc monetary incentives donated by enterprises. During the study period in May 2021 (i.e., three months since the vaccine rollout), the first-dose COVID-19 vaccination rate among Hong Kong residents aged ≥12 years was only about 20% [28]. It is warranted to understand factors of COVID-19 vaccination at this early vaccine rollout stage. As of 8 October 2022, 92.1% of the Hong Kong adults have completed the primary COVID-19 vaccination series [28].

Given the background, this study reported prevalence of completed or scheduled first-dose COVID-19 vaccination (CSFCV) in the Hong Kong adult general population aged 18 to 75 years old. Three categories of factors of CSFCV were investigated, i.e., (a) personal POE and personal NOE, (b) societal POE, and (c) prosociality. This study also investigated whether the association between societal POE and CSFCV would change regarding significance or magnitude after adjusting for personal POE. Furthermore, this study investigated whether prosociality would moderate the associations between the personal/societal outcome expectancy variables and CSFCV. Several hypotheses were tested in the present study: (1) the three groups of factors (i.e., personal POE/NOE, societal POE, and prosociality) would be significantly associated with CSFCV; the association involving NOE would be negative while the rest would be positive; (2) societal POE would have become non-significant (full mediation) or have a weaker association (partial mediation) after controlling for CSFCV via personal POE, and (3) the strength of the association between personal POE/NOE and CSFCV would be smaller at higher levels of prosociality.

## 2. Materials and Methods

### 2.1. Participants and Data Collection

A population-based telephone survey was conducted among the study population of adults aged 18–75 years of the general population in Hong Kong, China from 14–27 May 2021, which has a size of six million adults. The household fixed line telephone penetration rate was 78.2% in Hong Kong as of May 2022 [29]. A few measures were taken to reduce selection bias. First, simple random sampling (SRS) was used to select telephone numbers from an updated residential phone directory (i.e., the sampling frame); SRS involves high internal validity and external validity (i.e., better representativeness of the study population) [30] and is preferred over convenience sampling. Second, one member was selected from each household having at least one eligible member according to the birthday rule (the one whose birthday was closest to the survey time). Third, the survey took place between 5–10 pm to avoid over-sampling participants not engaging in work. Like any other studies, selection bias cannot be completely eliminated but reasonable measures had been exercised to reduce the bias. Unanswered telephone calls were given at least three attempts. Appointments were made if necessary. Trained interviewers briefed the participants about the objectives, content, and anonymity of the study, and the right to refuse to participate in the study or quit anytime without being questioned. They then sought verbal informed consent from the participants and signed a form pledging completion of the required consent procedures. As full addresses were not listed in the directory, it was not feasible to send invitation letters to the participants in advance. The same telephone survey method was used in other studies [31,32]. No incentives were given to the participants. The response rate, defined as the number of completed interviews (500) divided by the number of eligible contacts (880) was 56.8%, which is comparable to other telephone surveys [31,32]. Ethics approval was obtained from the Survey and Behavioral Research Ethics Committee of the Chinese University of Hong Kong (Ref No. SBRE-20-722).

### 2.2. Development of the Questionnaire

A panel comprising three public health experts was formed to develop the survey instrument. A thorough literature search was performed. As scales assessing personal POE/NOE and societal POE regarding COVID-19 vaccination were unavailable, items were generated by the panel following the guideline for developing items for the HAPA developed by Ralf Schaworzer [17], which has been used in many studies [14,15,33]. The guideline provides a definition for outcome expectancy: “(Outcome expectancies are) subjective beliefs about contingencies of an individual’s behavior with subsequent outcomes. These outcomes are evaluated with regard to their favorableness for the individual as positive or negative”. Following the guideline, the behavior of CSFCV in this study was defined in the first step. In Step 2, the items assessing the outcome expectancies were formulated. Items of the personal and societal domains were created by the panel accordingly, considering sample items given in the guideline and additional ones found in literature of vaccination studies [14,15,34]. The prosociality scale has been validated in other studies [35]. The questionnaire was tested in a pilot survey involving five participants to confirm comprehensiveness and was then finalized by the panel.

### 2.3. Measurements

#### 2.3.1. Background Information

Background information was collected, including sex, age, educational level, marital status, chronic disease status, and history of influenza vaccination.

#### 2.3.2. Completed or Scheduled First-Dose COVID-19 Vaccination (CSFCV)

A single item assessed whether participants had actually taken up at least one dose of COVID-19 vaccination, or made an appointment to take up the first dose COVID-19 vaccination (yes/no).

#### 2.3.3. Personal POE

There were four domains of perceived personal benefits. Good internal consistency was observed for the overall scale and the four subscales (Cronbach’s alpha ranged from 0.89 to 0.97). CFA was performed to test the unidimensional nature of the scales having more than two items; all results are satisfactory.

1. Physical benefit (protection): Three items assessed the levels of agreement with the statements that COVID-19 vaccination behavior could (i) effectively protect oneself from COVID-19 infection, (ii) effectively protect family members from COVID-19 infection, and iii) reduce the risk of developing severe harms and deaths, in the case of having COVID-19 infection (1 = totally disagree to 5 = totally agree; Cronbach’s alpha = 0.96; CFA results: χ^2^/*df* = 0, CFI = 1.00, TLI = 1.00, SRMR = 0.00).
2. Practical benefit: Four items assessed the levels of agreement with the statements that COVID-19 vaccination behavior could facilitate (i) visits to relevant public venues (e.g., restaurants and pubs), (ii) traveling with ‘vaccine passports’, (iii) fulfillment of the need/requirement related to work, and (iv) restoration of ‘normal social life’ (1 = totally disagree to 5 = totally agree; Cronbach’s alpha = 0.97; CFA results: χ^2^/*df* = 8.99, CFI = 0.99, TLI = 0.97, SRMR = 0.02).
3. Emotional benefit: Two items assessed the levels of agreement with the statements that COVID-19 vaccination behavior could relief oneself from worries about (i) COVID-19 infection, and (ii) severe harms or death in the case of having COVID-19 infection (1 = totally disagree to 5 = totally agree; Cronbach’s alpha = 0.95).
4. Interpersonal benefit: Two items assessed the levels of agreement with the statements that COVID-19 vaccination behavior could (i) remove social pressure when the participant’s friends ask about his/her COVID-19 vaccination status, and (ii) increase the participant’s friends’ willingness to have social gathering with him/her (1 = totally disagree to 5 = totally agree; Cronbach’s alpha = 0.89).
5. Summative scale of POE (the Overall Personal Positive Outcome Expectancy Scale; OPPOES): It was formed by adding up all the 11 aforementioned item scores (Cronbach’s alpha = 0.94; CFA results: χ^2^/*df* = 5.64, CFI = 0.95, TLI = 0.92, SRMR = 0.05).

#### 2.3.4. Personal NOE

One item assessed “Your COVID-19 vaccination uptake would incur severe side effects and even death” (1 = totally disagree to 5 = totally agree).

#### 2.3.5. Societal POE

Three items assessed the levels of agreement with the statements that COVID-19 vaccination behavior could help (i) controlling the COVID-19 outbreak in Hong Kong, (ii) facilitating prompt local economic recovery, and (iii) facilitating prompt removal of travel restrictions between Hong Kong and mainland China such as the quarantine requirements (1 = totally disagree to 5 = totally agree; Cronbach’s alpha = 0.97; CFA results: χ^2^/*df* = 0.00, CFI = 1.00, TLI = 1.00, SRMR = 0.00).

#### 2.3.6. Prosociality

It was assessed by using the 4-item Prosocial Behavioral Intention Scale, which has been validated and showed satisfactory psychometric properties among university students [35]. Its Chinese version has been applied to Chinese university students [24]. A sample item was “Comfort someone I know after they experience a hardship”. The items were rated with seven-point Likert scale (1 = definitely would not do this to 7 = definitely would do this); higher mean scores indicated higher levels of prosociality. The Cronbach’s alpha was 0.75 in this study.

### 2.4. Sample Size Planning

Sample size planning was conducted by using the Logistic Regression module of the PASS 11.0. Assuming the prevalence of CSFCV was 19% during the study period [28], the sample size of 500 yielded a smallest detectable odds ratio (OR) of 1.42 (power of 0.80 and alpha of 0.05, two-sided) when comparing two groups having a mean and a mean plus one standard deviation values in the independent variables of concern (e.g., personal POE).

### 2.5. Statistical Analysis

To allow for comparisons, the item mean scores of the various POE scales were obtained by dividing the summative scale scores by the number of items. Pearson correlation coefficients were derived to describe the correlations among the studied variables. Univariable and multivariable (adjusted for background factors) logistic regression analyses were conducted to test the associations between each of the independent variables and the binary dependent variable of CSFCV. Crude odds ratios (ORc), adjusted odds ratios (ORa), and respective 95% confidence intervals (CIs) were derived.

Path analysis was performed to test the mediation hypothesis that the association between societal POE and CSFCV via personal POE. It is an extension of multiple regression modeling, which can be used to analyze more complicated models. It allows a variable to serve as an independent variable of another variable and a dependent variable of a preceding variable simultaneously in a model. It can thus be used to mediations. Individual models were fit for every potential mediator (i.e., the overall scale and subscales of personal POE). The bootstrapping approach (n = 2000) was used to assess the mediation effects, which would be considered statistically significant when 95% CI did not include zero. The effect size of a mediation was calculated by indirect effect (i.e., a × b) divided by the total effect [i.e., (c’ + a × b)] [36]; a, b, and c’ refer to the effect of the independent variable (IV) on the mediator, the mediator on the dependent variable (DV), and the IV on the DV after adjusting for the mediator, respectively. A full mediation occurs when the IV becomes non-significantly associated with the DV after adjusting for the mediator; partial mediation is found when the effect of the IV on the DV is reduced in absolute size but remains statistically significant after adjusting for the mediator [36]. Conceptually, a full mediation means that the mediator can fully explain the association between the IV and the DV while a partial mediation means that the mediator has explained part but not all the association between the IV and the DV [36].

It is acknowledged that causal effects cannot be claimed in cross-sectional studies. It is hence clear that path analysis of cross-sectional data (including the present study) cannot be used to establish causality. Readers are reminded that only associations but not causality were generated in this study as the directions of the reported associations remain unclear (e.g., the temporal sequence is unknown). Notably, path analysis is made up of regression components [37], while regression analysis involves ‘predicted value” [38] and has been commonly used in published cross-sectional studies. Like regression analysis, path analysis has regularly been applied to a large number of cross-sectional studies [39,40,41,42,43], including the present one. Notably, some researchers have questions using path analysis in cross-sectional data [44,45], while others may be less critical [46,47]. Readers are strongly reminded to bear in mind that the aim of this study was to test whether the magnitude of the association between societal POE and COVID-19 vaccination would change after adjusting for the personal POE variables, instead of trying to find any causations, nor testing any theories. However, path analysis may still give readers some interesting formative insights. As a preliminary study and the first study of the type, the proposed mediation model only meant for hypothesis generation, which needs to be tested and confirmed by future longitudinal studies.

A moderation analysis was also performed to test the potential moderation effects of prosociality for the associations between personal POE and NOE/societal POE and CSFCV, using the Process Macro of SPSS. The model consists of the main effects of the IV (e.g., personal NOE) and the potential moderator (prosociality) in addition to an interaction term formed by multiplication of the IV and the moderator (e.g., personal POE × prosociality). The statistical significance of the interaction term denotes a significant moderation [36], implying that the strength of the association between the IV and the DV depends on the value of the moderator. In our case, it means that the strength of the associations between the POE/NOE variables and CSFCV would vary according to the level of prosociality.

All analyzed questionnaires have completed the survey. There were very few missing data as the interviewers were experienced in probing responses. In fact, there were no missing data in the key variables and the background variables, except that eight missing values were found in the question about educational level. To retain the cases for data analysis, a category of “missing response” was used when recoding the data in the logistic regression analysis. Mplus 7.0 was used to conduct path analysis while SPSS 23.0 was used for other statistical analyses. Statistical significance was defined as two-tailed *p*-value < 0.05.

## 3. Results

### 3.1. Descriptive Statistics

Participant characteristics were summarized in Table 1. The prevalence of CSFCV was 21.0%, including those having taken up at least one dose of COVID-19 vaccination (16.2%) and those having made an appointment to do so (4.8%).

The mean [standard deviation (SD); range] values were 3.1 (0.8; 1–5) for the OPPOES, 3.4 (0.9; 1–5) for physical benefit, 3.6 (0.9; 1–5) for practical benefit, 3.1 (1.0; 1–5) for emotional benefit, 2.5 (0.9; 1–5) for interpersonal benefit, 3.5 (1.0; 1–5) for personal NOE, 3.5 (1.0; 1–5) for societal POE, and 5.0 (1.1; 1–7) for the prosociality scale.

### 3.2. Correlation Analysis

The personal/societal POE variables were significantly and positively correlated with each other (*r* ranged from 0.49 to 0.87; *p* < 0.001). Personal NOE was negatively associated with all the personal/societal POE variables (ranged from −0.10 to −0.47; *p* < 0.05). The results are presented in Supplementary Table S1.

### 3.3. Factors of CSFCV

The ORc of all the independent variables were of statistical significance. Adjusted for the background factors, significant factors positively associated with CSFCV included (a) the overall scale of OPPOES [*ORa = 3.67 (95% CI: 2.58–5.22)*] and the four types of personal POE [*physical benefit: ORa = 2.91 (95% CI: 2.16–3.91); practical benefit: ORa = 2.45 (95% CI: 1.79–3.34); emotional benefit: ORa = 3.04 (95% CI: 2.27–4.07); interpersonal benefit: ORa = 1.65 (95% CI: 1.30–2.09)*] and (b) societal POE [*ORa = 2.11 (95% CI: 1.62–2.74)*]. Personal NOE was negatively associated with CSFCV [*ORa = 0.28 (95% CI: 0.21–0.38)*]. The association between prosociality and CSFCV was non-significant [*ORa* = *1.22 (95% CI: 0.99–1.51)*] but the non-significance was marginal as the *p*-value was only slightly larger than 0.05 (0.06); its ORc was statistically significant. The results are shown in the supplementary Table S2.

### 3.4. Mediation Analysis

In Table 2, it is seen that (1) the OPPOES, physical benefit, and emotional benefit fully mediated the association between societal POE and CSFCV; (2) practical benefit partially mediated the same association (mediation effect sizes of 49.2%); (3) interpersonal benefit did not significantly mediate the association. In other words, societal POE was associated with these POE/NOE variables, which were also significantly associated with CSFCV.

### 3.5. Moderation Analysis

**Figure 1:**
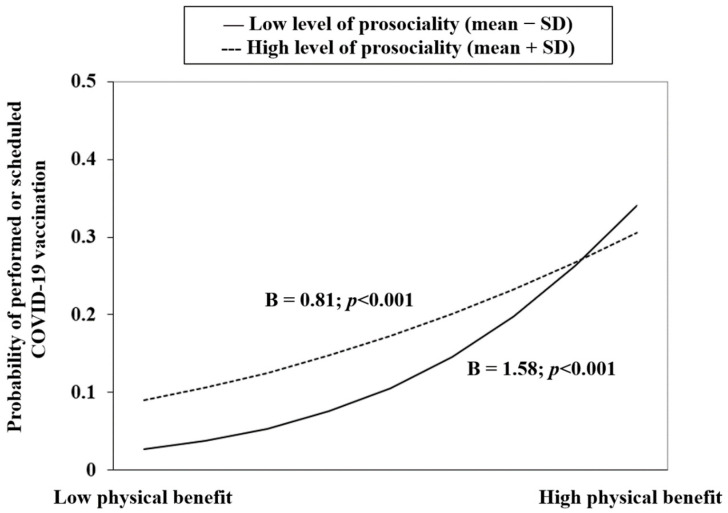
Prosociality moderated between physical benefit and CSFCV.

**Figure 2:**
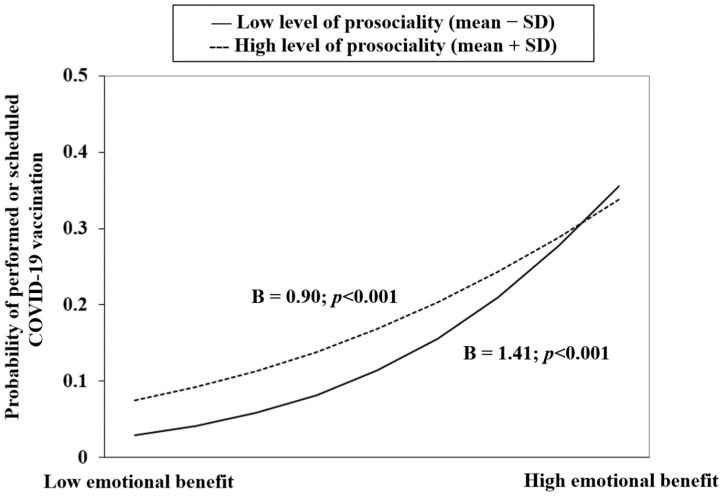
Prosociality moderated between emotional benefit and CSFCV.

In Table 3, prosociality significantly moderated the association involving the POE of physical benefit (i.e., physical benefit × prosociality; *p* = 0.009), but not the other associations. It is seen in Figure 1 that a stronger association between physical benefit and CSFCV was found in those having lower prosociality, while the effect of prosociality and CSFCV was stronger in those with lower level of physical benefit. Another interaction model (emotional benefit × prosociality) was marginally non-significant but showed a *p*-value slightly higher than 0.05 (0.073); its graphical presentation (Figure 2) is similar to Figure 1.

## 4. Discussion

The present study reports low prevalence of CSFCV of 21% in the Hong Kong adult general population, which was comparable to the 19.1% reported by the Hong Kong government as of 27 May 2021 (i.e., the last day of the study period) [28]. In the adjusted analysis, all the studied positive and negative outcome expectancies variables (personal POE/NOE and societal POE) were significantly associated with CSFCV. The association between societal POE and CSFCV was fully mediated via the OPPOES/physical benefit/emotional benefit and partially mediated via practical benefits but non-significantly via interpersonal benefit. The association between prosociality and CSFCV was marginally non-significant in the adjusted analysis, with a *p*-value of 0.06. It, however, significantly moderated the association between physical benefit and CSFCV; a similar marginally non-significant moderation for the association between emotional benefit and CSFCV was found, having a *p*-value of 0.073.

The levels of three types of POE (practical/physical/societal benefits) and that of personal NOE were moderately high. (1) Practical benefit yielded the highest mean, plausibly because the pandemic has disrupted daily life substantially [48], resulting in large resource losses (e.g., income, fun, and social relationships) [48]. (2) Understandably, physical benefit of protection exhibited a high mean, as previous studies have shown that perceived efficacy of COVID-19 vaccines has stronger impacts on vaccination decision than other attributes [11]. (3) Corroborating other studies [24,34], many participants believed that their COVID-19 vaccination behavior would result in societal benefits. (4) The comparably high level of NOE reminds us that POE and NOE were competing forces of similar magnitude affecting COVID-19 vaccination decisions. In contrast, the level of emotional benefit was relatively low. Although many studies have documented elevated mental distress during the pandemic [49,50], Hong Kong only reported on average <2 new COVID-19 cases per day during the study period [51] and might not have caused much strong mental distress. The level of interpersonal benefit was the lowest, suggesting relatively mild social pressure toward COVID-19 vaccination, possibly due to the low prevalence of COVID-19 vaccination and hence the absence of strong social norm.

The strengths of the associations involving POE and NOE were comparably strong, corroborating previous studies that perceived efficacy and safety of COVID-19 vaccines were strongly associated with COVID-19 vaccination [11]. A novel finding is that practical benefits and emotional benefits were significantly associated with CSFCV. Practical incentives may have played significant roles in accelerating vaccination; linkages between vaccination and entry to venues/service utilization/travel had/have been established in many countries (e.g., the U.S., the U.K., and Australia) to boost vaccination rates. Emotions may be involved in COVID-19 vaccination. Societal POE was also significantly associated with CSFCV; enhancement of such has been used to promote vaccination [21,22,52].

It is also novel to report a significant association between the trait of prosociality and CSFCV. Its role on CSFCV is, however, inconclusive as a positive but marginally non-significant association (*p* = 0.06) was found with relatively small sample size. Literature also presented mixed findings of both significant [24,27] and non-significant [53] associations between prosociality and COVID-19 vaccination intention. Furthermore, prosociality significantly moderated the association between physical benefit and CSFCV, i.e., physical benefit seemed less important in affecting CSFCV in those with high prosociality than those with low prosociality. It is plausible that individuals with high prosociality might rely less on self-directed motives when making vaccination decisions. From another angle, the impact of prosociality on CSFCV became stronger when physical benefit became lower.

Another novel finding of this study is that the associations between societal POE and CSFCV became non-significant after adjusting for the overall scale or the two subscales of personal POE (physical benefit and emotional benefit) individually. A hypothesis that societal POE may lead to an increase in personal POE, which would in turn increase COVID-19 vaccination, was generated by these findings but required confirmation in future longitudinal studies.

This study has hence contributed to literature and filled out some important knowledge gaps. Some novel findings have been reported. First, it has extended our knowledge about the associations between perceived benefits and COVID-19 vaccination; this study included various types of personal benefits and societal benefits, which were significantly associated with CSFCV. Second, it found the mediation mechanism of the association between societal POE and CSFCV via personal benefits, i.e., societal POE was associated with personal POE, which was in turn associated with CSFCV. Third, prosociality significantly moderated the association between perceived physical benefits and CSFCV. Thus, the study sheds insights on new research directions and has widened angles that can be applied to understanding the relationships between expected positive and negative impacts regarding vaccination. Future longitudinal studies may investigate mediators between POE/NOE and vaccination and other COVID-19 related behaviors and test SCT and HAPA theories.

This study also has some potential practical implications. Outcome expectancies are modifiable and health promotions modifying perceived outcomes of vaccination have shown to be effective [54]. First, health promotion programs promoting vaccination should modify POE and NOE simultaneously as they may have competing impact of similar strengths on CSFCV. Second, both practical benefits (e.g., entry to venues/service utilization/travel) and emotional benefits should be emphasized as both were significant factors of CSFCV. Third, promotion should publicize both personal and societal benefits; this “dual benefits” approach has been used to promote vaccination [21,22,52]; increase in societal POE would increase personal POE according to the mediation effect. Fourth, prosociality should be elevated. Fifth, as prosociality moderated the association between physical benefit and CSFCV, it is important to promote physical benefit of vaccination among those who are less prosocial. As the study was based on cross-sectional associations, readers are reminded that the afore-mentioned implications could only be useful if future longitudinal research could confirm the present study’s findings.

It is a limitation that data of this study were collected during the early phase of the vaccine rollout in Hong Kong when the vaccination rate was relatively low. It is, however, important to record factors of COVID-19 vaccination at various phases of the vaccine rollout period. Although some countries have currently attained relatively high vaccination rates and are promoting booster doses, factors identified at early phases remain valuable. First, such factors may have contributed to the eventual increase in vaccination rates. Second, identification of such factors may inform countries lagging behind in their vaccination rates. Third, it may facilitate promotion of novel vaccines when future pandemics occur. In literature, similar factors of COVID-19 vaccination and vaccine hesitancy (e.g., perceived benefits) have been found consistently in studies conducted across different phases of vaccine rollout [55,56]. It is hence expected that the factors found in this study may still be applicable to scenarios involving higher vaccination rates and booster vaccination. In particular, the roles of self-directed and others-directed motives of COVID-19 vaccination investigated in this study are quite general in nature, and should be applicable across phases of vaccine rollout, although confirmation is required.

The present study has other limitations. First, as mentioned, temporal or causal inferences cannot be made due to the cross-sectional nature of this study. Confirmation in longitudinal studies is warranted. Second, social desirability bias for taking up COVID-19 vaccination might exist. Third, although the response rate of this study (56.8%) was comparable to those of previous local telephone surveys [31,32], participants and non-participants may have different characteristics but such information was not available. Fourth, although the age distribution of this study [(aged 18–50: 56.0% (sample) versus 51.2% (census); aged 51–75: 44.0% (sample) versus 48.8% (census)] was in general comparable to that of the 2021 Hong Kong census data, female sex [60.6% (sample) versus 52.7% (census)] may have been slightly over-represented in this sample. Fifth, people aged >75 years were not included in this study, as their decisions for COVID-19 vaccination might be affected by their health status; it is unclear whether the factors of CSFCV would be the same in this age group as those of the younger age groups. Sixth, the sample size was relatively small, some associations with *p*-values slightly above 0.05 (e.g., prosociality) may become statistically significant if the sample size were larger. Seventh, the study sample involved Hong Kong Chinese and generalization of the results to other Chinese population would require cautions due to differences in social and epidemiological contexts. Eighth, this study is preliminary and other mediators (mechanisms) between outcome expectancies and COVID-19 vaccination have not been tested. Ninth, this study was conducted during a specific vaccine rollout period; the findings might differ from those studies conducted in other vaccine rollout phases. Lastly, it is an important limitation of this study that scales assessing personal POE/NOE and societal NOE were not available. Hence these scales had not been evaluated in other studies. We have generated Cronbach’s alpha to assess internal consistency and CFA to confirm the one-dimensionality of the scales but it was not feasible to validate these scales in the same study as such an exercise would require an independent sample. Future validation of an outcome expectancy scale for COVID-19 vaccination is greatly warranted.

## 5. Conclusions

The objectives of this study were to find out the prevalence of CSFCV and its associations with various types of POE/NOE and prosociality and to test related mediation and moderation hypotheses. The prevalence of CSFCV among Hong Kong adults at three months since vaccine rollout was about 20%. This study has three novel findings. First, both various types of personal POE/NOE and societal POE were significantly and strongly associated with CSFCV. Second, the association between societal POE and CSFCV was fully mediated by some personal POE scales. Third, the association between prosociality and CSFCV was marginally non-significant; prosociality, however, moderated the association between physical benefit and CSFCV. Health promotion programs tend to focus on promoting physical benefits of vaccination (e.g., protection from infection and reduction in severe disease outcomes) and reducing perceived harms of COVID-19 infection. The findings remind health workers to widen the scope of health promotion to include other types of POE such as practical and emotional benefits. It is equally important to pay attention to promoting societal POE, which was also strongly associated with CSFCV. Promotion of societal POE has another advantage as it might increase POE which might in turn increase CSFCV, according to the findings of the significant mediation model. The findings also give support to theories that involve POE/NOE (e.g., SCT and HAPA) and encourage the use of such theories to predict COVID-19 vaccination and related interventions. Longitudinal research is required to confirm the findings in settings of high and low vaccination rates and across countries. It is worthy to conduct pilot randomized controlled studies investigating the efficacy of interventions modifying comprehensive types of personal POE/NOE as well as societal POE.

## Figures and Tables

**Table 1:** Participants’ characteristics (n = 500).

	n	%
**Background factors**		
Sex		
Female	303	60.6
Male	197	39.4
Age groups (years)		
18–50	280	56.0
51–75	220	44.0
Educational attainment		
Below college	353	70.6
College or above	139	27.8
Missing response	8	1.6
Chronic disease status		
No	370	74.0
Yes	130	26.0
History of influenza vaccination		
No	373	74.6
Yes	127	25.4
**Completed or scheduled first-dose COVID-19 vaccination (CSFCV)**		
No	395	79.0
Yes	105	21.0

**Table 2:** Path analysis testing the mediation effect of personal POE between societal POE and CSFCV (n = 500).

Mediators	IV > M	M > DV	IV > DV	Indirect Effect	Effect Size
	*β*	*β*	*β*	*β (95% CI)*	
OPPOES	0.83 ***	0.57 ***	−0.08	0.48 (0.32–0.63)	Full
Physical benefit	0.80 ***	0.50 ***	−0.01	0.40 (0.27–0.53)	Full
Practical benefit	0.76 ***	0.26 **	0.20 *	0.19 (0.07–0.32)	49.2%
Emotional benefit	0.64 ***	0.48 ***	0.09	0.31 (0.22–0.39)	Full
Interpersonal benefit	0.59 ***	0.07	0.35 ***	0.04 (−0.03–0.11)	NS

Note. IV = Independent variable (societal POE in this case); M = Mediator; DV = Dependent variable (completed or scheduled first-dose COVID-19 vaccination in this case; CSFCV); CI = Confidence interval; POE = Positive outcome expectancy; OPPOES = The Overall Personal Positive Outcome Expectancy Scale; NS = Non-significant as the 95% CI included zero. *β:* Standardized coefficients were reported. * *p* < 0.05; ** *p* < 0.01; *** *p* < 0.001. The models were adjusted for age, sex, educational level, chronic disease status, and history of influenza vaccination.

**Table 3:** Moderation analysis testing the moderation effect of prosociality between personal/societal outcome expectancies and CSFCV (n = 500).

	Completed or Scheduled First-Dose COVID-19 Vaccination (CSFCV)	
	ORa (95% CI)	*p*
*Model 1*		
OPPOES	10.61 (1.97–57.01)	0.006
Prosociality	2.42 (0.78–7.45)	0.124
OPPOES × Prosociality	0.81 (0.60–1.11)	0.190
*Model 2*		
Physical benefit	18.87 (4.24–83.92)	<0.001
Prosociality	4.26 (1.46–12.42)	0.008
Physical benefit × Prosociality	0.70 (0.54–0.92)	0.009
*Model 3*		
Practical benefit	2.31 (0.56–9.48)	0.244
Prosociality	1.15 (0.39–3.40)	0.806
Practical benefit × Prosociality	1.01 (0.77–1.32)	0.946
*Model 4*		
Emotional benefit	10.51 (2.51–44.03)	0.001
Prosociality	2.55 (0.98–6.66)	0.055
Emotional benefit × Prosociality	0.79 (0.61–1.02)	0.073
*Model 5*		
Interpersonal benefit	1.90 (0.63–5.78)	0.256
Prosociality	1.37 (0.74–2.55)	0.319
Interpersonal benefit × Prosociality	0.98 (0.79–1.21)	0.836
*Model 6*		
Personal NOE	0.44 (0.12–1.57)	0.206
Prosociality	1.38 (0.65–2.96)	0.405
Personal NOE × Prosociality	0.92 (0.72–1.17)	0.502
*Model 7*		
Societal POE	4.73 (1.31–17.10)	0.018
Prosociality	2.17 (0.83–5.68)	0.114
Societal POE × Prosociality	0.86 (0.68–1.08)	0.192

Note. ORa = Adjusted odds ratio; CI = Confidence interval; OPPOES = The Overall Personal Positive Outcome Expectancy Scale; NOE = Negative outcome expectancy; POE = Positive outcome expectancy. The models were adjusted for background factors, including sex, age groups, educational level, chronic disease status, and history of influenza vaccination.

## Data Availability

The data presented in this study are available on request from the corresponding author. The data are not publicly available due to ethical considerations.

## Supplementary Materials

The following are available online at https://www.mdpi.com/article/10.3390/vaccines10111883/s1, Table S1: Correlation among the personal/societal outcome expectancy variables. Table S2: Factors of completed or scheduled first-dose COVID-19 vaccination.
