# Supplementary material for: How Motives Related to Benefits for Oneself and Others Would Affect COVID-19 Vaccination in a Hong Kong Chinese General Adult Population?
Source: Vaccines (Basel). 2022 Nov 8;10(11):1883. doi: 10.3390/vaccines10111883 (PMC9698433; doi:10.3390/vaccines10111883)
Supplement: Supplementary file 1 [file vaccines-10-01883-s001.zip › vaccines-1908210-supplementary.pdf]

Supplementary Material

Table S1. Correlation among the personal/societal outcome expectancy variables (n = 500)

	1	2	3	4	5	6
**Personal POE**						
1. OPPOES	-					
2. Physical benefit	0.87***	-				
3. Practical benefit	0.85***	0.65***	-			
4. Emotional benefit	0.83***	0.70***	0.57***	-		
5. Interpersonal benefit	0.80***	0.55***	0.64***	0.49***	-	
**6. Personal NOE**	−0.34***	−0.32***	−0.22***	−0.47***	−0.10*	-
**7. Societal POE**	0.83***	0.79***	0.75***	0.65***	0.59***	−0.24***

Note. POE = Positive outcome expectancy; OPPOES = The Overall Personal Positive Outcome Expectancy Scale; NOE = Negative outcome expectancy. *, p <0.05; **, p< 0.01; ***, p < 0.001.

Table S2. Factors of completed or scheduled first-dose COVID-19 vaccination (n = 500)

	Completed or scheduled first-dose COVID-19 vaccination (CSFCV)			
	ORc (95% CI)	*p*	ORa (95% CI)	*p*
**Personal POE**				
OPPOES	3.31 (2.38-4.60)	<0.001	3.67 (2.58-5.22)	<0.001
Physical benefit	2.78 (2.09-3.70)	<0.001	2.91 (2.16-3.91)	<0.001
Practical benefit	2.25 (1.68-3.00)	<0.001	2.45 (1.79-3.34)	<0.001
Emotional benefit	2.81 (2.15-3.69)	<0.001	3.04 (2.27-4.07)	<0.001
Interpersonal benefit	1.57 (1.26-1.97)	<0.001	1.65 (1.30-2.09)	<0.001
**Personal NOE**	0.30 (0.22-0.39)	<0.001	0.28 (0.21-0.38)	<0.001
**Societal POE**	1.95 (1.53-2.50)	<0.001	2.11 (1.62-2.74)	<0.001
**Prosociality**	1.25 (1.03-1.53)	0.026	1.22 (0.99-1.51)	0.060

Note. ORc = Crude odds ratio; ORa = Adjusted odds ratio; CI = Confidence interval; POE = Positive outcome expectancy; OPPOES = The Overall Personal Positive Outcome Expectancy Scale; NOE = Negative outcome expectancy. The models were adjusted for background factors, including sex, age groups, educational level, chronic disease status, and history of influenza vaccination. The range of personal and societal outcome expectancies were 1-5; their ORs were hence comparable.
